# Evaluating the utility of a novel 3D mobile imaging application in the surgical management of craniosynostosis

**DOI:** 10.1007/s00381-026-07124-z

**Published:** 2026-01-15

**Authors:** Emery Buckner-Wolfson, Geena Jung, Hailey Reisert, Margaret Keymakh, Timothy Kim, Genesis Liriano, Oren Tepper, Andrew Kobets

**Affiliations:** 1 Department of Neurosurgery, Albert Einstein College of Medicine, Bronx, NY USA; 2 Department of Neurosurgery, Montefiore Medical Center, Bronx, NY USA; 3 Department of Plastic and Reconstructive Surgery, Montefiore Medical Center, Bronx, NY USA

**Keywords:** Craniofacial, Craniosynostosis, Metopic, Trigonocephaly, Cranial vault reconstruction, Stereophotogrammetry, Mobile application

## Abstract

**Purpose:**

Craniosynostosis is one of the most common craniofacial defects. It is typically treated with surgical intervention to correct abnormal head shape. Currently, there is no standard method for evaluating treatment effectiveness. We aim to assess the utility of MirrorMe3D, a novel three-dimensional mobile imaging application, in monitoring the head shape changes of two patients with craniosynostosis.

**Methods:**

We implemented the application in the surgical management of a 22-month-old with mild metopic craniosynostosis and a 19-month-old with severe trigonocephaly. Using the application, scans were taken preoperatively, intraoperatively, and postoperatively. Three-dimensional models were generated from these scans then compared qualitatively through visualization of the skull contour and quantitatively using depth and volume analysis features of MirrorMe3D.

**Results:**

The application was easily integrated into the surgical workflow. Scanning and generating models took no more than 5 min. Comparing preoperative and postoperative models, we found a 22% volume reduction and a depth change of 3.01 mm in the metopic ridge of the 22-month-old and a depth change of 3.12 mm in the area of interest for the 19-month-old. The models also provided a clear way to visualize changes of the skull contour for clinicians and families.

**Conclusion:**

MirrorMe3D provides an efficient and inexpensive method of evaluating changes in head shape by generating 3D models and enabling quantitative measurements. Not only can these images contribute to intraoperative decision-making and inform the current family about changes made during surgery, but they can show future families what to expect and help guide decision-making.

**Supplementary Information:**

The online version contains supplementary material available at 10.1007/s00381-026-07124-z.

## Introduction

Craniosynostosis is a developmental craniofacial anomaly caused by the premature fusion of one or more cranial sutures. It affects approximately 1 in every 2000 to 2500 live births worldwide and can be spontaneous, syndromic, or familial [7, 11, 16]. The early closure of cranial sutures causes restriction of skull growth perpendicular to the affected suture and compensatory growth parallel to that suture, resulting in various cranial malformations, including scaphocephaly, plagiocephaly, trigonocephaly, and brachycephaly [24]. In severe cases, craniosynostosis can lead to increased intracranial pressure, neurodevelopmental delay, cognitive deficits, visual impairments, and other serious complications [2, 10, 29]. The deformity may also have psychosocial implications later in life. Thus, early diagnosis and treatment are imperative to mitigate these complications. Due to the heterogenous etiologies and clinical presentations, treatment is highly variable. Most treatment options involve surgical intervention, which may be elective for solely cosmetic reasons or may be necessary to ensure normal brain development [12].

Due to the wide variety of treatment options, it is essential to evaluate the effectiveness of each method to help clinicians and families make informed decisions. However, there is currently no established method of quantitatively measuring treatment effectiveness. Although computed tomography (CT) is utilized for diagnostic purposes and surgical planning in craniosynostosis cases, it is generally avoided postoperatively due to cost, sedation, and the importance of avoiding further exposure to harmful ionizing radiation [14, 18, 20]. Instead, treatment outcomes are typically evaluated qualitatively based on provider and family assessments of the change in head shape and positioning of facial features, which is a subjective analysis, and with clinical measurements such as cephalic index (CI), cranial vault asymmetry index (CVAI), and head circumference, which are prone to interobserver variability and do not capture the full surface morphology of the skull [15]. Recently, noninvasive three-dimensional imaging techniques have been emerging to fill this gap. These include 3D lasers, light optical scans, and photogrammetry techniques [20].

However, the existing technologies that employ these imaging modalities have many drawbacks. Most of them involve stationary, bulky, and expensive equipment that is difficult to integrate into the operating room (OR) and requires highly trained personnel to manipulate. The mobile imaging application, MirrorMe3D, provides a safe, efficient, and accessible alternative. It implements stereophotogrammetry to generate 3D models from scans taken by the user. It is inexpensive, easily downloaded on a mobile device, requires minimal training to operate, and may be used in many settings. The user simply takes a series of photos of the patient and the app generates a 3D model. Subsequent quantitative analyses can be performed with the length, angle, depth, volume, and surface area measurement features of the MirrorMe3D platform and multiple models can be compared. The app can also be downloaded by patients and their families, enabling quick and easy data sharing with providers for remote monitoring of patient progress.

Here, we aim to assess the applicability of this technology in the surgical management of craniofacial abnormalities by evaluating its ease of use, integration into the OR, and utility in evaluating qualitative and quantitative changes in head shape after treatment of two patients with craniosynostosis. We hypothesize that it will not significantly disrupt the surgical workflow and that it will provide useful intraoperative and postoperative assessments of head shape for both clinicians and families to better guide management of craniofacial defects.

## Methods

This study was approved by Albert Einstein College of Medicine’s Institutional Review Board, and the researchers have no financial relationship with MirrorMe3D.

### Case presentations

The legal guardians of the two patients provided informed consent for participation in this study and use of patient images.

Patient 1 was a 22-month-old male who was diagnosed with craniosynostosis with a metopic ridge without overt trigonocephaly. Although current practice is to observe such benign pathology, the patient’s parents expressed concern about his appearance and desired to pursue surgical correction of the forehead protrusion, so a procedure to shave down the metopic ridge was planned.

Patient 2 was a 19-month-old who was diagnosed with trigonocephaly. Surgical correction was recommended due to overt disfigurement. He was neurologically normal and meeting developmental milestones. The patient’s family opted to move forward with a full cranial vault reconstruction.

Due to the diagnostic certainty of both cases and to avoid unnecessary radiation exposure, neither of the patients underwent CT imaging prior to or following the procedures.

### Procedures

Patient 1 underwent a metopic ridge shaving. A midline incision in the hairline was made down to the pericranium, which was then dissected off the bone down to the nasion. Using an endoscope and alternating with a lighted retractor, the ridge was shaved down from the fontanelle to the level of the nasion using a coarse 3-mm drill. After achieving a normal contour, the wound was closed in standard fashion using absorbable sutures on the skin.

Patient 2 underwent an open anterior cranial vault remodeling with an interdisciplinary team of neurosurgeons and plastic surgeons. First, a bicoronal incision was opened down to the pericranial layer. Meticulous work was done to take the pericranium off the calvarium and elevate it as a separate layer. Burr holes were placed posteriorly on either side of the midline sinus and bilaterally temporally. Flo Seal and a Penfield one dissector were used to take the dural attachments off of the underside of the calvarium. An M2 drill bit was used to make the burr holes, and a B5 attachment was used to elevate the bone. Then, using a Marchac template, a semicircular piece of bone was cut from the elevated craniectomy to be used as the new forehead. The bandeau was isolated using cuts at the orbital roof, nasofrontal junction, and temporal bone. After all the bone segments were removed, the neo-forehead, cut from the Marchac template, was reshaped then attached to the bandeau using endocortical absorbable plates. This piece was secured to the temporal bone using more absorbable plates and a midline sagittal strut. A suture lattice network was used to create full coverage with the remaining bone fragments from the midline strut to the temporal bones laterally on either side. Finally, the pericranial layer and skin were replaced and sutured over the bone construct, and the wound was closed using absorbable sutures.

### Intraoperative scanning

At various points during the operation, authorized research personnel trained in operating the technology took scans of the patient’s head on a company-issued iPhone with the MirrorMe3D application. For each scan, research personnel took photos at 8 different orientations around the patient’s head, with approximately 4 photos per orientation, starting overhead and moving down incrementally to just above profile, level with the patient’s head, and below profile level. Since the photos were taken by moving the phone around the patient’s head with no tripod or other equipment, the same research personnel took scans at each time point to standardize the scanning technique. Lighting was kept constant across each scan. After the scans were captured, 3D models were generated and saved in the app.

For Patient 1, one scan was taken preoperatively after fiducial points were marked on the patient’s head to use as reference points for maintaining consistency across scans. A second scan was taken postoperatively following closure.

**Fig. 1:**
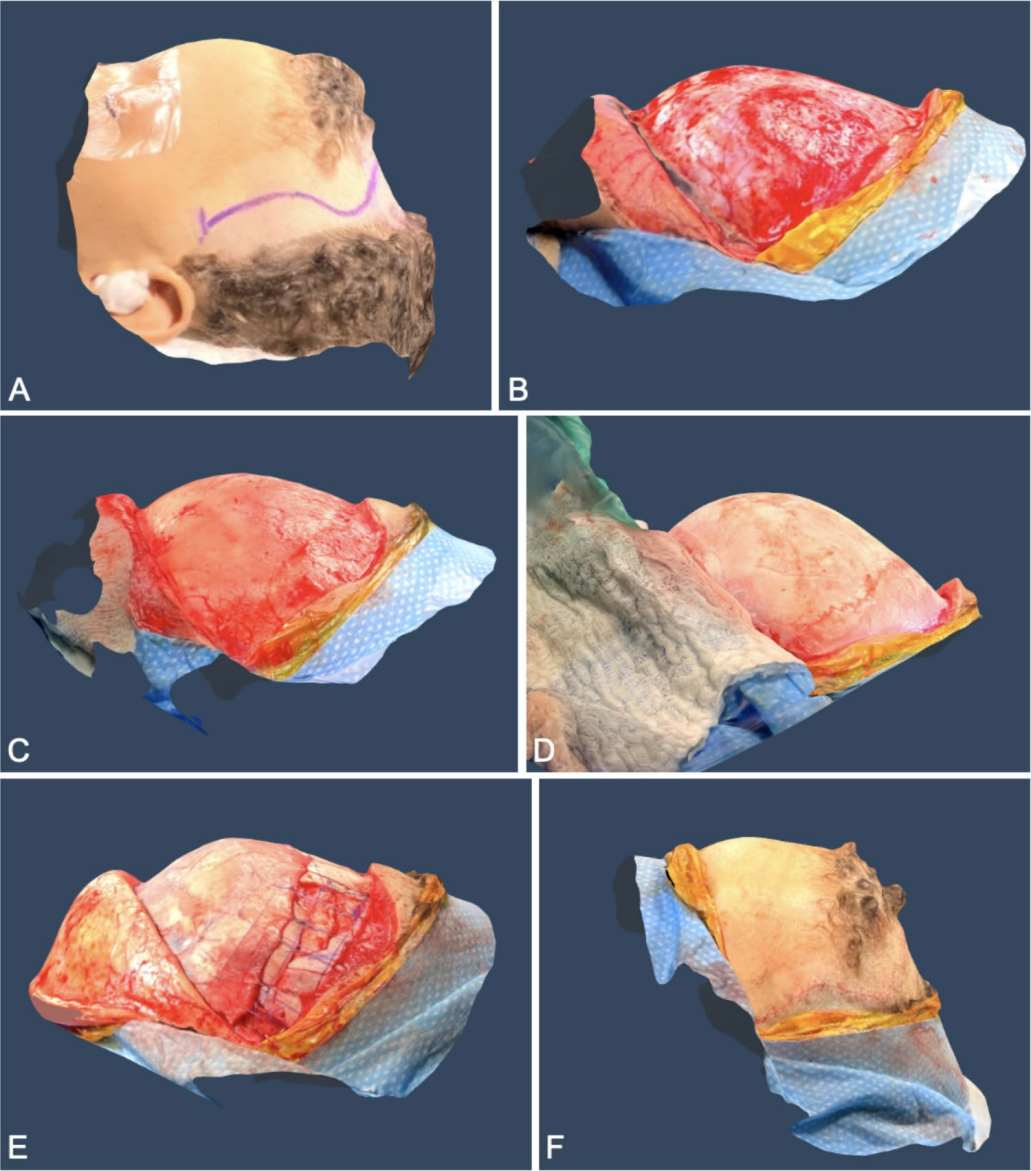
Profile view of intraoperative scans from Patient 2. **A** Preoperative scan after the patient’s hair was shaved and the incision line was marked on the scalp. **B** Intraoperative scan following incision with skin retracted exposing the pericranium. **C** Intraoperative scan after dissection and retraction of the pericranial layer to expose the cranium. **D** Intraoperative scan after bone removal exposing the dura mater. **E** Intraoperative scan after bone remodeling. **F** Postoperative scan following suture closure

For Patient 2, a total of seven scans were taken throughout the procedure at each stage: (1) preoperative scan prior to prepping, (2) preoperative scan after prepping, (3) intraoperative scan following incision with skin retracted exposing the pericranium, (4) intraoperative scan after dissection and retraction of pericranial layer to expose the cranium, (5) intraoperative scan after bone removal exposing the dura, (6) intraoperative scan after bone remodeling, and (7) postoperative scan following closure (Fig. 1).

### MirrorMe3D validation and storage of scans

MirrorMe3D has completed extensive bench testing to confirm that the system accurately reflects input data upon display. The scans are stored as Digital Imaging and Communications in Medicine (DICOM) files on the MirrorMe3D platform through the data storage provider Amazon Web Services. The platform automatically converts DICOM files to Nearly Raw Raster Data format files for 3D display. There were no other pre- or postoperative imaging taken for this study because the treatment team did not want to expose the patients to unnecessary radiation.

### Data analysis

Data analysis was conducted by the online MirrorMe3D platform, which allowed for visual comparisons of the 3D models generated throughout the operations. To measure change in head shape, preoperative and postoperative models were aligned using four fiducial points, and the area of interest on the forehead was isolated. The change in depth and volume was measured from this isolated area. First, the depth changes in the overlaid models were visualized using a heat map feature of the MirrorMe3D platform. Then, the change in depth was calculated for both patients, and volume change was measured for the metopic ridge in Patient 1. There was no comparison to other imaging modalities performed in the current study to avoid unnecessary exposure to radiation.

## Results

The MirrorMe3D application generated 3D models of the patient’s head from the operating table and above. These models were viewed and rotated by the user within the mobile app as well as on the desktop platform (see Supplementary Video).

### Intraoperative scanning

Intraoperative scans were successfully obtained in both cases. The acquisition time for scans was approximately 3 min, and the number of photos included in each scan ranged from 30 to 40. The process of generating the three-dimensional models from these scans took approximately 2 min; however, it was highly dependent on internet connectivity. The average processing speed was 2–3 s per photo, but this increased with poor connectivity and with a greater number of photos included.

### Image analysis

**Fig. 2:**
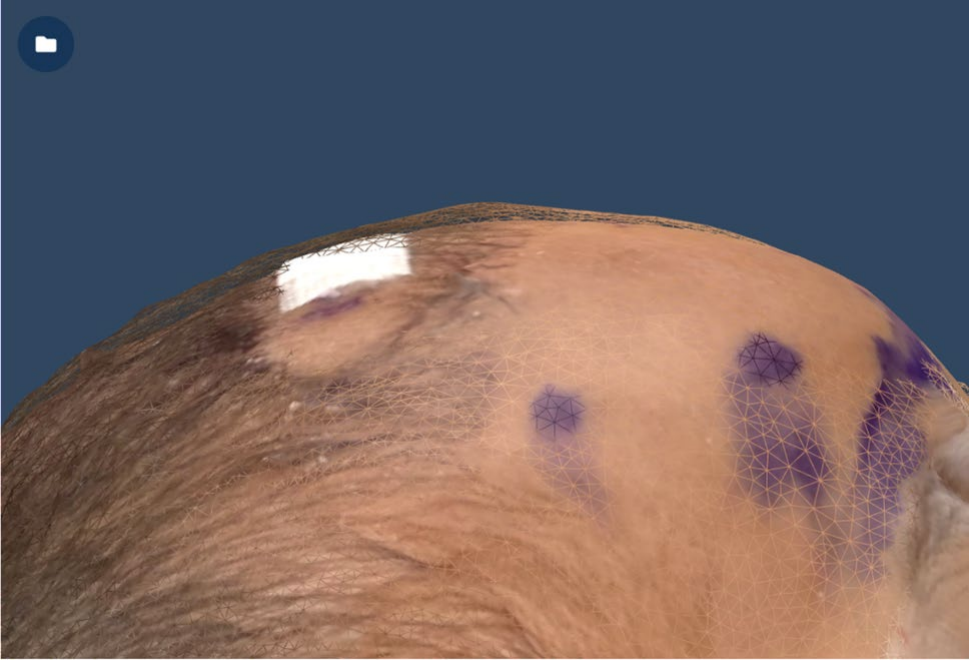
Overlay of the preoperative skull contour (mesh layer on top) and the postoperative skull layer (underlying pigmented layer), demonstrating a reduction of the metopic ridge of Patient 1 following surgery

**Fig. 3:**
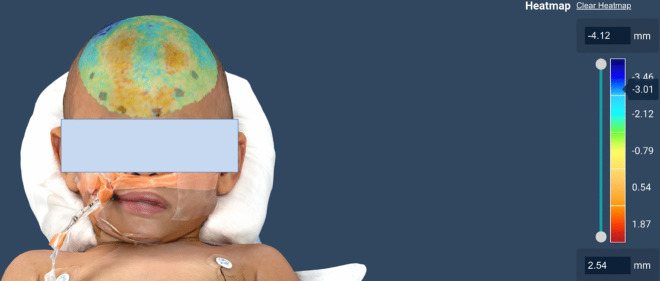
Heat map of the overlayed models from Patient 1, showing the depth change between the preoperative and postoperative skull contours. The largest calculated depth difference in the area of interest is 3.01 mm

For Patient 1, the preoperative and postoperative models were aligned to visualize the reduction of the metopic ridge (Fig. 2), and the greatest calculated depth difference in the isolated area of interest was 3.01 mm (Fig. 3). The preoperative volume of the area of interest was measured at 152 cc and the postoperative region was 118 cc, so there was an overall volume reduction of approximately 22%.

**Fig. 4:**
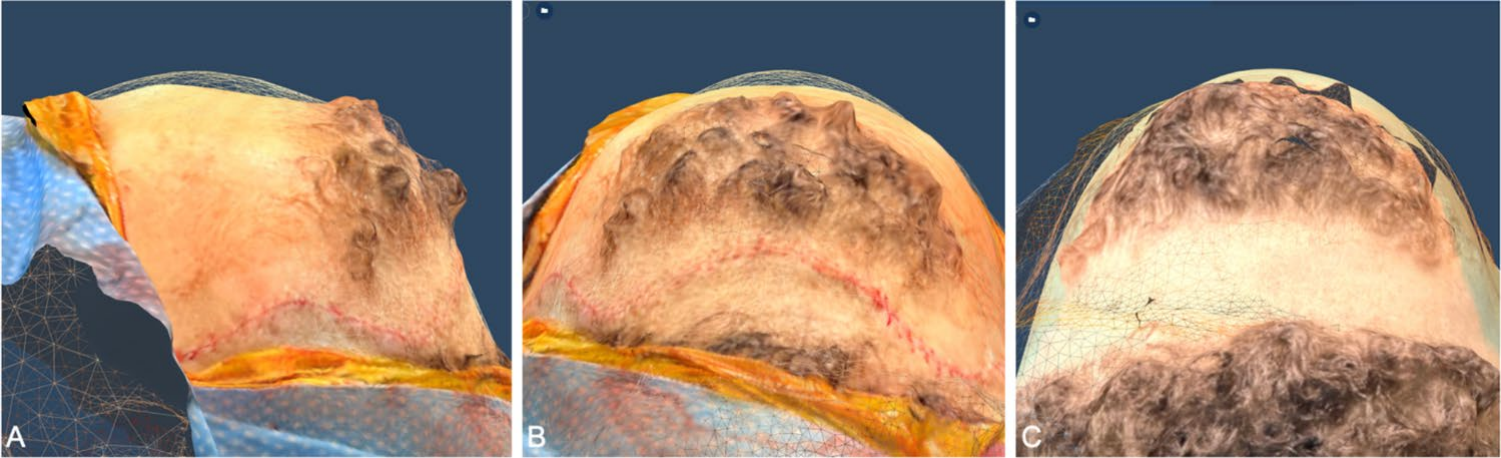
Overlay of preoperative and postoperative skull contour for Patient 2. **A** Profile view showing reduction of metopic ridge and change in skull contour (preoperative model is mesh layer on top and postoperative is pigmented layer below). **B** View from the top of the head showing a flattening of the skull (preoperative model is mesh layer on top and postoperative is pigmented layer below). **C** View from the top of head showing the widening of the head (postoperative model is mesh layer on top and preoperative is pigmented layer below)

**Fig. 5:**
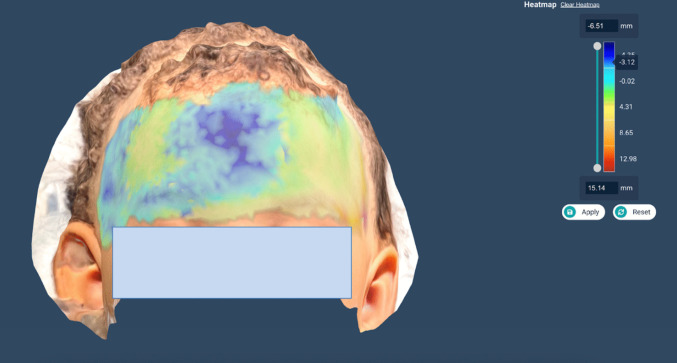
Heat map of the overlayed models from Patient 2, showing the depth change between the preoperative and postoperative skull contours. The largest calculated depth difference is 3.12 mm

For Patient 2, the second preoperative scan was aligned with the postoperative scan, and the overlaid models were used to visualize the changes in the skull contour (Fig. 4). The greatest calculated depth difference in the isolated area of interest was 3.12 mm (Fig. 5).

## Discussion

Craniosynostosis is a commonly seen condition in pediatric neurosurgery that has been increasing in prevalence [6, 28]. There are many different clinical presentations, predisposing factors, and underlying etiologies of craniosynostosis that factor into the complex management of patients. Most treatment options involve surgical intervention to correct abnormal head shape and prevent complications.

Currently, there is a lack of consensus on which treatment options provide the most optimal outcomes because there is no standardized method for objectively quantifying the severity of deformity before and after treatment [22]. Thus, evaluation of treatment effectiveness is often a subjective matter. Traditionally, direct anthropometric measurements using calipers and tape measures have been used to assess cranial deformity. However, these measurements have demonstrated low reliability and high inter-observer variability [25]. Moreover, they do not reflect the three-dimensional topography of the skull. The gold-standard imaging modality used for evaluation of head shape in craniosynostosis is CT. However, CT is rarely performed postoperatively due to the need for sedation in young children and the exposure to radiation leading to increased risk of cancer later in life [3–5]. Instead, clinicians and researchers have been turning to 3D photography as a noninvasive, reliable, and accurate method of assessing head shape in patients with craniofacial abnormalities like craniosynostosis.

Three-dimensional photography has been steadily rising in popularity [8]. Some of the existing technologies include 3D camera systems that employ photogrammetry techniques such as the 3dMD Cranial System, 3D laser scanners such as the M4D scanner, and 3D optical light scanners such as FaceScan3D. Countless studies have confirmed the validity, accuracy, precision, and reliability of these technologies compared to direct anthropometric measurements and measurements calculated from radiologic images [1, 17, 21–23, 25, 27, 30–32]. Multiple studies have used 3D photography to calculate pre- and postoperative cephalometric measurements like head circumference, cephalic index, volume, and angles [19, 30, 31], while other studies have implemented these technologies in combination with principal component analysis (PCA), statistical shape modeling, and machine learning to visualize head shape variations [22, 25, 26].

These technologies have numerous advantages over previously used methods. They are quick, more cost-effective, independent of the examiner, can be used in various settings, and do not require sedation or radiation. However, they also present challenges. Many of them involve stationary equipment with a multiple camera setup that is not feasible in the OR. They are costly, not widely available, and require highly trained personnel to operate, so most health care facilities do not have access to them. Even with the development of newer handheld devices like those reviewed in Knoops et al. [13], there are still multiple pieces of expensive equipment required, including cameras, lights, sensors, and computers. They also require cables connecting the device to a computer for processing purposes. García-Mato et al. found that the handheld structured light scanner, Artec Eva, was easily integrated into surgical workflow, but the range of motion of the user was limited by the cables [9]. Furthermore, all the existing technologies require multiple different hardware and software systems to capture, store, and analyze the images, which prolongs and complicates data processing.

The mobile imaging application used in the present study addresses many of these challenges. It is compact, portable, cheap, widely accessible, and easy to use, requiring minimal training. Additionally, each step of the process from scanning to storage to analysis is consolidated into a single software package. Here, we evaluated the utility of MirrorMe3D in the surgical management of two patients with craniosynostosis. The app was successfully used to take scans intraoperatively with little disruption to surgical workflow, and the 3D models generated from these scans enabled real-time evaluation of the skull contour at multiple stages throughout the operation. This could aid in intraoperative planning by helping surgeons assess whether the changes made are satisfactory or if further changes are necessary. Analysis of the models demonstrated a depth change of 3.01 mm and a 22% volume reduction in the metopic ridge of Patient 1 and a depth change of 3.12 mm in the forehead of Patient 2. The overlaid models also allowed for excellent visualization of the skull contour alterations, which helped demystify the process for the patients’ families. All the scans were archived on the MirrorMe3D platform for future use by clinicians and families.

Although MirrorMe3D has many advantages over the previously discussed technologies, it does not come without limitations. The scanning and processing of scans took approximately 5 min, which may not seem significant but can add up during surgery, when time must be optimized. Additionally, this time was highly dependent on internet connectivity, which may be lacking in the OR. Another drawback is that movement disrupts the platform’s ability to produce accurate reconstructions. While this is not an issue with adult patients, it poses a challenge in the pediatric population. Although this was circumvented in the present study because the patients were anesthetized, future implementation into the clinic or at home for continued monitoring will necessitate advancements in the technology and creativity on the part of the user. It is also important to note that the volume, surface area, and depth measurements as well as the alignment of two models for comparison require manual selection of points from which to calculate metrics and overlay models. This introduces the potential for human error. Finally, the calculated depth differences reported here were smaller than expected and this could have been due to errors in alignment or possibly the presence of swelling around the surgical site. Later postoperative follow-up imaging to allow for the reduction in swelling would be necessary to investigate this further.

We must also acknowledge the limitations of the study itself. First and foremost, it only involved two patients, which may call into question its reliability and applicability to other cases. Another drawback is the lack of comparison imaging such as pre- or postoperative CT scans, which could have provided additional measurements and data to compare to the measurements obtained from the app. Additionally, this technology was designed to avoid the use of additional equipment like tripods, which is ideal in the OR and contributes to ease of use but also impacts the standardization of the scanning protocol because research personnel used a freehand technique to capture the scans, introducing variability. The aforementioned limitations can be attributed to the nature of this study as a preliminary investigation of a novel application for a new technology. The successful implementation of the technology into the surgical management of these two patients allows for further investigations into its utility that will address these shortcomings. In current and future studies, we plan to include more patients with other pathologies involving craniofacial abnormalities such as plagiocephaly, fibrous dysplasia, and cephalohematoma. We will include additional imaging studies and clinical measurements to compare to the measurements obtained using the app, and we will continue to explore the other analysis capabilities of the MirrorMe3D platform such as angle and surface area measurements to further enhance our ability to assess head shape alterations.

Despite the limitations, it is clear that the MirrorMe3D app has the potential to greatly enhance the management of patients with craniosynostosis. It provides an accessible, inexpensive, efficient, and harmless method of quantitatively evaluating treatment effectiveness by generating 3D models, allowing for topographic visualization and comparison. It can be used as a tool for providers, patients, and family members to assess craniofacial deformity, guide intraoperative correction of the deformity, evaluate surgical outcomes, and help future health care teams make informed decisions.

## Conclusions

The MirrorMe3D application is a promising new technology that provides unique benefits to the clinical management of craniosynostosis. Not only is it easily integrated into the OR, generating real-time visuals that can help guide surgical correction, but it provides quantitative measurements of changes in head shape that can help clinicians and families evaluate treatment effectiveness and make informed clinical decisions. However, it is not without limitations, and further research must be conducted to ensure reliability and reproducibility. In the future, we hope to extend the use of this technology to other pathologies and settings, further enhancing clinical care of young patients with craniofacial abnormalities.

## Supplementary Information

Below is the link to the electronic supplementary material.

ESM 1 MP4 (114 MB)

## Data Availability

No datasets were generated or analysed during the current study.
